# Assessing compassionate abilities: Translation and psychometric properties of the Italian version of the compassionate engagement and action scales (CEAS)

**DOI:** 10.1371/journal.pone.0326922

**Published:** 2025-07-15

**Authors:** Amanda Nerini, Camilla Matera, Anna Rosa Donizzetti, Daniela Caso, Chiara Rollero, Caterina Grano, Duccio Baroni, Ptarmigan Plowright, Paul Gilbert

**Affiliations:** 1 Department of Education, Languages, Intercultures, Literatures and Psychology, University of Florence, Florence, Italy; 2 Department of Humanistic Studies, University of Naples Federico II, Naples, Italy; 3 Department of Psychology, University of Turin, Turin, Italy; 4 Department of Psychology, University of Rome La Sapienza, Rome, Italy; 5 IPSICO - Institute of Behavioral and Cognitive Psychology and Psychotherapy, Florence, Italy; 6 Centre for Compassion Research and Training, College of Health, Psychology and Social Care, University of Derby, Derby, United Kingdom; 7 The Compassionate Mind Foundation, c/o University of Derby, Derby, United Kingdom; Universiti Sains Malaysia - Kampus Kesihatan, MALAYSIA

## Abstract

This study aimed to develop the Italian version of the Compassionate Engagement and Action Scales (CEAS) and examine its validity and reliability among Italian-speaking adults. A total of 374 (mean age = 23.11) Italian speaking participants took part in the study. All of them completed a questionnaire comprising the CEAS, together with measures of self-compassion, self-criticism, social support, empathy, well-being and general distress, used to estimate the scale’s convergent and criterion-related validity. Confirmatory Factor Analysis (CFA) revealed a satisfactory fit for a model in which three second-order factors (Self-compassion, Compassion for others and Compassion from others) were further articulated in two first-order factors (Engagement and Action). All the scales presented good reliability in terms of internal consistency. Correlations with measures of social support, empathy, self-compassion, self-criticism, well-being, and general distress indicated good convergent and criterion-related validity of the Italian version of the CEAS. Taken together, these results suggest that the CEAS can be properly used with Italian-speaking individuals in order to assess the three compassion flows in terms of both engagement and action.

## Introduction

According to Gilbert [1], compassion is a basic motivation and like all motivations is rooted in a stimulus-response algorithm. The stimuli that activate the psychophysiological processes of caring are indicators of distress, suffering and need that trigger responses appropriate to that distress and need. In this model, it is important to always distinguish the competencies for being sensitive and understanding and engaging with suffering and the motives and competencies for appropriate action. Hence, compassion can be defined as 1. a sensitivity to suffering in self and others with 2. A commitment to try to alleviate and prevent this suffering [1,2]. The authors proposed an evolutionary approach to compassion, focusing on motivations and competencies, in which compassion can be experienced for oneself, can be received from other people and can be experienced for others. According to this perspective, compassion can flow in three directions: compassion individuals can direct towards themselves, compassion they feel for other people, and compassion individuals can receive from others [1,2].

Compassion directed to oneself, known as self-compassion, encourages a sensitivity and recognition of aspects of distress, suffering or need, the courage and wisdom to engage, with empathic insight into what is likely to be helpful and then to act appropriately. Individuals can be sensitive but not know what to do, or fail to act appropriately. Self compassion can be used to support ourselves in times of setback and disappointment and as a counter to self-attacking, but it can also be used in times of great distress such as receiving a diagnosis of cancer or losing someone we love [1,2]. Compassion from others refers to the capacity to receive but also to be appropriately responsive to compassion from other people and to feel soothed or encouraged and energised, when confronted by life’s difficulties. Knowing and turning to others who are available to help us is significantly related to coping with life difficulties [3]. Compassion for others refers to being open and moved by the suffering of others, recognizing that all humans are vulnerable to many sources of suffering in life and can also be sources of harm to self and others [1,2,4].

As noted, each of these three flows of compassion consists of two dimensions: engagement, which pertains to attention paid to suffering and signals of distress, and compassionate action, which refers to behaviours aimed at preventing and alleviating suffering. Engagement requires specific competencies and skills [2,5,6], such as motivation, attention sensitivity, sympathy, the ability to tolerate distress, empathic insight, and being non-judgemental in the condemning sense. Compassionate actions are characterized by paying attention to the things that can help, alleviate or prevent suffering, thinking and reasoning, behaving to address suffering, and being willing to experience a range of feelings and emotions [2].

### The three flows of compassion and health outcomes

While much research has indicated that compassion directed to oneself is associated with numerous indices of well-being and health, such as lower levels of depressive symptoms and anxiety [2,7,8], it is only in recent years that research has begun to focus on the effects of compassion experienced from others and for others on health-related outcomes.

The ability to perceive others as compassionate (namely, compassion from others) has been found to have a crucial role in reducing loneliness and improving quality of life [9], to be associated to happiness, self-esteem, and several indicators of affective and cognitive well-being [10], and to have a significant impact on health and resilience to distress [2,4]. Interestingly, being open and receptive to support, care, and kindness from others buffers the depressogenic effect of self-criticism, plays a protective role against the impact of major life events and represents an adaptive strategy to manage stressful circumstances [11]. Cunha et al. [12] showed that the impact of self-reassurance on life satisfaction was partially mediated by compassion from others. Hermanto and Zuroff [13] found that being open to the compassion from others buffers the effect of self-criticism on depression. Such data highlight the fact that to understand how compassion manifests in the world we need to focus on both competencies for giving *but also receiving* compassion from others.

Compassion for others implies the ability to detect signals of distress in other people, tolerate uncomfortable feelings that might emerge, connect to others without judging them, and be ready to do something to alleviate this observed suffering [2]. Some empirical evidence suggests that both desiring to be helpful to other people [14] and being able to express compassionate love for others [15] are positively associated with health.

The three flows of compassion are significantly interrelated, and they all contribute to individuals’ well-being. Matos et al. [16] found that the three compassionate flows were associated with lower psychological distress and higher social safeness during the COVID-19 pandemic across different countries. In a test of social mentality theory, Hermanto et al. [17] showed that high caregiving along with the ability to receive care predicted self-compassion, whereas high care-giving with low care-seeking (being less open and receptive to compassion) predicted poor self-compassion.

### The compassionate engagement and action scales (CEAS)

To date, only one instrument has been developed to measure the three compassionate abilities. This instrument is the Compassionate Engagement and Action Scales (CEAS), which was developed by Gilbert and colleagues [2] to assess Compassion for self, Compassion for others, and Compassion from others through three different scales. In line with the S-R algorithm of the motive of caring [18], each flow has two subscales: one to measure people’s sensitivity, tolerance and engagement with distress and suffering (i.e., Engagement) and one to measure the competencieies for taking appropriate actions aimed at alleviating and preventing distress and suffering (i.e., Action).

The original CEAS was validated in English and Portuguese in three samples of university students (American, British and Portuguese, respectively) [2]. The hypothesized factor structure of the scale was confirmed. The results suggested that the three compassionate abilities could be either assessed as three single-factor scales or divided into two different scores capturing engagement and action, respectively. The self-compassion engagement scale presented two factors: sensitivity to suffering and engagement with suffering. The CEAS showed acceptable levels of reliability and good convergent validity. The three compassionate orientations showed a moderate correlation with each other.

Following these first versions, the CEAS was adapted to other sociocultural contexts, such as Japan [19], France [20], Portugal [12], Sweden [21], Slovakia [22], Ecuador [23], Turkey [24], and Canada [25]. Validity and reliability of the CEAS were tested for the Japanese [19], Portuguese [12], Swedish [21], Slovak [22], Turkish [24], and Canadian [25] versions (see Table 1).

**Table 1:** Validity and reliability of the different versions of the CEAS.

Version	Factor structure	Deleted items	Reliability (internal consistency)	Reliability (test-retest)
Japanese [19]	Mono-factorial structure of each of the three scales	Compassion for others: item 4; Self-compassion: item: item 2, 4, and 8	Cronbach’s alphas between.84 and.92 for the three scales	Intraclass correlations between.67 and.72
Portuguese [12]	Two-factor structure for each of the three scales	Compassion for others: item 4	Cronbach’s alphas between.84 and.94 for the three scales	Intraclass correlations between.97 and.98
Swedish [21]	Two-factor structure for each of the three scales	Item 4 of each scale	Cronbach’s alphas between.74 and.92 for the three scales	Intraclass correlations between.77 and.85
Slovak [22]	Two-factor structure for each of the three scales	None	Cronbach’s alphas between.70 and.89 for the three scales	Not calculated
Turkish [24]	Two-factor structure for each of the three scales	None	Cronbach’s alphas between.70 and.95 for the six subscales	Not calculated
Canadian [25]	Self-compassion: three factor structure; two-factor structure for the Compassion for others and the Compassion from others scales	None	Cronbach’s alphas between.87 and.96 for the three scales	Not calculated

The dimensionality of the Japanese version [19] was tested among undergraduate students through Exploratory Factor Analysis (EFA), which revealed a mono-factorial structure of each of the three scales (Self-Compassion, Compassion for others, Compassion from others); some items (item 4 of the compassion for others scale; items 2, 4, and 8 of the self-compassion scale) were deleted because of their low factor loading on the corresponding factor. The Japanese version presented good reliability and convergent validity.

The CEAS was also validated with adolescents in both Sweden and Portugal. The Swedish adolescent version (CEASY-SE) [21] presented the same two-factor structure for each of the three scales (Compassion for self, Compassion for others, Compassion from others), although one item (item 4) had to be removed in each scale due to an insufficient correlation with the total score. Internal consistencies were good to excellent for the three scales. The three dimensions of compassion were significantly but not strongly related to one another. The factor structure of the Portuguese version for adolescents (CEAS-A) [12] was similar to the one found in the adults’ version, with higher-order factors encompassing two factors in each scale (Engagement and Actions). In line with the Swedish version, item 4 also created some problems within the Portuguese Compassion for Others scale and was therefore removed from it (but not from the other scales). The CEAS-A revealed good construct validity and reliability.

The Slovak version of the CEAS was validated with a sample of Slovak men and women [22]. The authors examined the dimensionality of this version by testing different models through different statistical analyses: the Slovak version was found to have a global factor representing each flow of compassion as a whole, and two specific factors representing Engagement and Action respectively. The two subscales within each of the three flows of compassion were found to have good internal consistency when they were considered to be unidimensional. When the Engagement dimension of the Compassion for others scale was split into two partial subscales, its reliability decreased considerably.

More recently, a Turkish version of the CEAS was developed and validated among college students [24]. A Confirmatory Factor Analysis (CFA) was performed separately on the three scales (Self-Compassion, Compassion for others, Compassion from others); a first-order two-factor model was found to have a good fit to the data for each scale. Items 4 and 8 presented low factor loadings on the Self-compassion scale; nevertheless, the authors decided to retain these two items, as they assess some aspects of self-compassion (the ability to embrace one’s distressed feelings) that should be considered from the respondent’s point of view. The Turkish version produced good reliability and convergent and divergent validity.

In 2024, Brophy and colleagues [25] validated the French version of the CEAS, testing its psychometric properties with French-Canadian participants. The CFA did not completely support the structure of the original version of the CEAS. Specifically, the Compassion for self scale showed a three-factor structure, while the Compassion for others and the Compassion from others scales presented a two-factor structure. Internal consistency was good for all subscales except from the Sensitivity to suffering subscale of the Self-compassion scale. The CEAS was invariant across age and presented good convergent validity.

In sum, the CEAS has good psychometric properties and has been widely used by clinicians and researchers around the world. In all contexts in which the CEAS was adapted, the three scales showed high reliability and good convergent validity. In some contexts the Self-compassion scale presented a more complex structure, articulated in three rather than two components. In some adaptations item 4 emerged as problematic and was either deleted or maintained.

### The present study

In Italy, the most used scale for assessing self-compassion is the Italian version [26] of the Self-Compassion Scale [27] based on a bipolar conceptualization of this construct. It consists of three positive (self-kindness, common humanity, and mindfulness) and three negative (self-judgment, isolation, and over-identification) components [28]. However, there are no scales assessing compassion either for or from others and there is not a comprehensive scale that measures compassion in terms of both engagement and action. The availability of a validated, Italian translation of the CEAS would significantly help advance research and intervention related to compassion in this national and linguistic context. Based on these considerations, the present study aimed to develop the Italian version of the CEAS and examine its psychometric properties among Italian-speaking adults.

After translating the scale items, we performed the CFA in order to assess the factorial validity of the CEAS.

Both convergent and criterion-related validity were examined by calculating correlations with various constructs previously used in validation studies of the CEAS, including social support, empathy, self-compassion, self-criticism, well-being, and general distress. With respect to convergent validity, the following associations were hypothesized. Given that compassion for oneself implies the ability to reassure oneself, the Self-compassion scale of the CEAS was hypothesized to correlate moderately and positively with the tendency to be self-reassuring, while it was hypothesized to be negatively and moderately correlated with self-criticism. It was also predicted to be moderately and positively correlated with the Self-Compassion Scale by Neff [27]. The Compassion from others scale was hypothesized to be positively and moderately correlated with perceived social support from different sources, given that compassion from others is conceptually related to the recognition of support from other people. The Compassion for others scale, which assesses both noticing distress signals or indicators of suffering in other people, and empathic connection with others’ suffering, was hypothesized to be positively and moderately correlated with empathy.

With regard to criterion-related validity, in line with the findings obtained by Gilbert and colleagues [2], we assumed that the three compassion scales would be positively related to participants’ well-being, with Compassion for others displaying a weak correlation, while Self-compassion and Compassion from others would show a moderate correlation. Self-compassion and Compassion from others were also assumed to be weakly and negatively correlated with general distress.

## Materials and methods

### Study design

We adopted a cross-sectional design to test the Italian CEAS validity and reliability.

### Study setting and participants

Participants (n = 374) were recruited through various strategies, including advertisements on social networking sites and recruitment from different university courses (mainly given within Psychology Schools) in Central, Northern and Southern Italy. Our target population were female and male adults. The only inclusion criterion was being of age. Only participants who did not provide their written informed consent were excluded. No other exclusion criteria were established. Participation in the study was voluntary and anonymous. Personal data were never asked to participants, so that their privacy and security was completely granted. Participants’ place of residence, employment, education and age are presented in Table 2.

**Table 2:** Participant demographic characteristics (N = 374).

		n	%
Gender	Female	269	71.9
	Male	99	26.5
	Other	6	1.6
Place of residance	Southern Italy and islands	156	41.7
	Central Italy	123	32.9
	Northern Italy	91	24.3
	Other	4	1.1
Education	primary school degree	3	0.8
	High school	282	75.4
	Bachelor’s degree	52	13.9
	Master’s degree	29	7.8
	postgraduate degree	8	2.1
Employment	university students	262	70.1
	workers	100	26.7
	unemployed	8	2.1
	Retired	3	0.8
	homemakers	1	0.3
Age	from 18 to 70 years (M = 23.11, SD = 7.63).		

### Data collection

Data were collected between 1 March and 31 May 2024. Data are available on OSF (https://osf.io/xw3bt). Participants who agreed to take part in the study were first asked to give their written informed consent to participate. If they provided this, the survey continued, otherwise it ended with a message of thanks. The survey was administered via the Google Forms platform and was completed in approximately 25 minutes. Some participants completed the survey at the end of a lesson, while some others provided their answers in different moments directly assessing the survey through the provided link. There were no missing responses in the dataset.

### Measures

Participants completed a questionnaire comprising the following measures.

**Compassionate Engagement and Action Scales (CEAS).** In order to adapt the CEAS into Italian, we followed broadly accepted translation guidelines [29]. First of all, three independent Italian researchers fluent in English forward-translated the CEAS instructions, items, and response options from English to Italian. Then, one independent researcher reviewed and resolved any minor inconsistencies. An independent bilingual translator back-translated the Italian version into English. Finally, the two translations were compared by an expert committee comprising psychologists and researchers with considerable expertise in this topic. Minor inconsistencies between versions were solved through discussion. This final version was approved by the first author of the original version of the CEAS (for the Italian version of the CEAS see Appendix).

The CEAS [2] is a 39-item scale with a 10-point Likert response format (1 = never; 10 = always). It comprises three scales: Self-compassion, Compassion from others, and Compassion for others. Each subscale consists of two components: Engagement (8 items, e.g., Self-compassion: “I tolerate the various feelings that are part of my distress”; Compassion from others: “Others notice and are sensitive to my distressed feelings when they arise in me”; Compassion for others: “I tolerate the various feelings that are part of other people’s distress”) and Action (5 items, e.g., Self-compassion: “I take the actions and do the things that will be helpful to me”; Compassion from others: “Others treat me with feelings of support, helpfulness and encouragement”; Compassion for others: “I take the actions and do the things that will be helpful to others”). In all three scales, there are some filler items that are not used to compute the scale score; they are items 3 and 7 of each Engagement subscale and item 3 of each Action subscale. Higher scores represent greater levels of compassion. Internal consistency scores of the three subscales are reported in the Results section.

**Self-compassion.** Self-compassion was measured through the Italian version [26] of the Self-Compassion Scale [27], which is composed of 6 subscales: Self-kindness, assessing an attitude of care and understanding toward oneself in the face of suffering; Self-judgment, measuring the tendency to disapprove and harshly judge one’s flaws and inadequacies; Common humanity, measuring perception of personal experiences of failure as elements shared by humanity; Isolation, assessing the perception of being the only one suffering or making mistakes; Mindfulness, which measures a state of awareness, attention, and acceptance of one’s negative thoughts and feelings in a balanced and nonjudgmental way; Over-identification, referring to the excessive identification with and absorption in one’s feelings and emotions. The Self-Compassion scale is a 26-item scale with a 5-point Likert-type response format (1 = almost never; 5 = almost always) with six factors: Self-kindness (one’s capacity to be kind and understanding with oneself, e.g., “I’m tolerant of my own flaws and inadequacies”) versus Self-judgment (the inclination to be excessively critical towards oneself, e.g., “I’m intolerant and impatient towards those aspects of my personality I don’t like”); Common humanity (the ability to see one’s suffering and failures as part of human nature, e.g., “When I’m down and out, I remind myself that there are lots of other people in the world feeling like I am”) versus Isolation (the feeling of being separated from the rest of human experience, e.g., “When I fail at something that’s important to me I tend to feel alone in my failure”); Mindfulness (the ability of holding painful thoughts and feelings in balanced awareness, e.g., “When something upsets me I try to keep my emotions in balance ”) versus Over-identification (an excessive immersion in and identification with one’s own feelings and emotions, e.g.,“ When I’m feeling down I tend to obsess and fixate on everything that’s wrong”). Higher levels of self-compassion correspond to higher scores on self-kindness, common humanity, and mindfulness, and lower scores on self-judgment, isolation, and over-identification. The internal consistency for this scale in the present study was good (α = .92).

**Self-criticism and self-reassuring.** The Italian version [30] of the Forms of Self-criticizing/attacking and the Self-reassuring Scale (FSCRS) [31] were used to evaluate one’s typical reaction to an event when something goes wrong, according to a self-critical or self-reassuring style. The FSCRS is a 22-item scale with a 5-point Likert from 0 (not at all like me) to 4 (extremely like me) composed of three subscales, including two forms of self-criticism, namely Inadequate-self and Hated-self, and one of self-reassuring, namely Reassured-self. The Inadequate-self involves feelings of inadequacy and a sense of frustration toward one’s self (e.g., “There is a part of me that feels I am not good enough”). The hated self refers to a more extreme form of self-criticism including the desire to persecute the self (e.g., “I have a sense of disgust with myself”), and the Reassured-self refers to the ability to treat the self with kindness and supportiveness when facing faults and failures (e.g., “I am able to remind myself of positive things about myself”). The internal consistency for this scale in the present study was good (Inadequate-self, α = .90; Hated-self, α = .81; Reassured-self, α = .87).

**Empathy.** Empathy was measured through adaptation of four items rated on a 5-point Likert from 1 (not at all) to 5 (a lot) developed by Capozza and colleagues [32] (e.g., “To what extent, when you think about other people, do you share their emotions?”). Higher scores represented greater levels of empathy. The internal consistency for this scale in the present study was good (α = .76).

**Social support.** Perceived social support was assessed through the Italian version [33] of the Multidimensional Scale of Perceived Social Support (MSPSS) [34]. The scale consists of 12 items ranging from 1 (very strongly disagree) to 7 (very strongly agree) divided into three subscales depending on the source of support: perception of support from family (e.g., “I can talk about my problems with my family”), friends (e.g., “My friends really try to help me”) and from significant others (e.g., “There is a special person who is around when I am in need”). Higher scores represented greater levels of perceived social support. The internal consistency for this scale in the present study was good (α = .90).

**Psychological wellbeing.** We used the Italian short-form version [35] of the Ryff’s Psychological Wellbeing Scale [36] to assess participants’ psychological well-being. This 18-item version of Ryff’s Psychological Well-Being Scale (PWB) is composed of 18 items (e.g., “When I look at the story of my life, I am pleased with how things have turned out”), which are rated on a 6-point Likert-like scale (from 1 = definitively disagree, to 6 = definitively agree). High scores indicate high levels of well-being. The internal consistency for this scale in the present study was good (α = .81).

**General distress.** General distress and negative emotions were assessed through the Italian version [37] of the short form of the Depression Anxiety Stress Scales-21 (DASS-21) [38]. The scale consists of 21 items ranging from 0 (Did not apply to me at all) to 3 (Applied to me very much, or most of the time) measured three factors: Depression (e.g., “I could see nothing in the future to be hopeful about”); Anxiety (e.g., “I was worried about situations in which I might panic and make a fool of myself”) and Stress (e.g., “I found it difficult to relax”). Higher scores represented greater levels of general distress. The internal consistency for this scale in the present study was excellent (α = .95).

### Statistical analyses

Before testing the factorial structure of the Italian version of the Compassionate Engagement and Action Scales (CEAS) by Gilbert and colleagues [2], we conducted a preliminary analysis of the data collected using IBM SPSS Statistics (version 29). We looked at item distributions in order to identify items with too high skew (> |2|) and/or kurtosis (> |7|) values; based on commonly accepted guidelines, data can be considered normally distributed when having skewness between −2 and +2 and kurtosis between −7 and +7 [39]. The analysis of internal consistency, for the identification of scale items that are not consistent with each other, was verified through the analysis of the corrected item-to-scale correlation indices (or item-total correlation), the scores of which must be greater than.30 in absolute value [40]. Reliability was tested in terms of internal consistency of the scales, by means of Cronbach’s alpha coefficient, the values of which are judged inadequate if below.60, satisfactory if between.60 and.69, good if between.70 and.79, very good if between.80 and.89, excellent if equal to or greater than.90 (Nunnally and Bernstein, 1994).

The next step was to verify the factorial structure of the CEAS through CFA using the M-PLUS 8.0 software [41]. The Chi-squared distribution and the degrees of freedom (Chi-squared/df ≤ 3) were used to assess the deviation between the reproduced and observed matrix, although this index is sensitive to sample size. Reference was also made to the following indices: Standardised Root Mean Square Residual (SRMR ≤ 0.09); Comparative Fit Index (CFI > 0.90); and Tucker-Lewis index (TLI > 0.90). If the results of the Root Mean Square Error of Approximation (RMSEA) are ≤ 0.05, they are considered to be good, and reasonable if they are ≤ 0.08, with a cut-off value of 0.06. To assess the goodness of fit of the model, several indices will be considered simultaneously since the different indices assess different aspects of goodness of fit [42–45]. We used a theory-driven approach to define our model. Nevertheless, modification indices were used as an important tool in order to find misspecifications and identify indicators’ error terms that had a high correlation [46]. These were allowed to covariate only if they were related to similarly worded items [47].

Evidence of convergent and criterion-related validity was collected via bivariate Pearson correlation coefficients. A correlation between.1 and.3 was considered small, between.3 and.5 was medium, and above.5 was considered large [48].

### Ethical considerations

The study was designed in line with the ethical standards of the APA, the ethical principles of the 1995 Declaration of Helsinki and was approved by the local ethics committee (Ethics Committee of the University of Turin, prot. 0639477 del 07/12/2023).

## Results

### Descriptive analyses

Table 3 shows the variances, means, standard deviations, skewness, and kurtosis of the CEAS items, as well as the corrected item-total correlations. The results indicate that the data were normally distributed (skewness < 1.77, kurtosis < 3.24), so no correction to fit indices was necessary. All item-test correlations were between 0.41 and 0.69, which suggests good psychometric properties.

**Table 3:** Mean, Standard deviations, Variance, Asymmetry, Kurtosis, and corrected correlations of the item.

	*Mean*	*SD*	*Variance*	*Skewness*	*Kurtosis*	*Corrected item-total correlations*
*Self-compassion Engagement*						
Item 1	6.23	2.416	5.838	−0.377	−0.804	0.57
Item 2	7.56	2.214	4.902	−0.916	0.018	0.59
Item 4	7.14	2.455	6.029	−0.772	−0.441	0.47
Item 5	5.63	2.417	5.843	−0.152	−0.961	0.52
Item 6	7.15	2.488	6.189	−0.883	−0.179	0.58
Item 8	5.34	2.611	6.816	0.080	−1.045	0.41
*Self-compassion* *Action*						
Item 1	6.59	2.169	4.704	−0.489	−0.432	0.59
Item 2	6.45	2.214	4.902	−0.462	−0.442	0.59
Item 4	6.46	2.035	4.142	−0.418	−0.354	0.60
Item 5	5.89	2.464	6.069	−0.295	−0.849	0.52
*Compassion for others Engagement*						
Item 1	7.82	1.948	3.794	−1.270	1.402	0.62
Item 2	8.18	1.826	3.334	−1.541	2.420	0.67
Item 4	7.29	2.307	5.320	−0.726	−0.388	0.42
Item 5	7.01	2.129	4.531	−0.824	0.096	0.56
Item 6	7.79	2.011	4.042	−1.262	1.167	0.62
Item 8	7.61	2.457	6.039	−1.045	0.051	0.41
*Compassion for others Action*						
Item 1	7.82	1.940	3.765	−1.365	1.898	0.64
Item 2	7.88	1.864	3.475	−1.280	1.695	0.66
Item 4	7.38	1.943	3.775	−0.944	0.802	0.59
Item 5	8.43	1.819	3.307	−1.770	3.240	0.66
*Compassion from others Engagement*						
Item 1	5.89	2.145	4.601	−0.243	−0.779	0.69
Item 2	5.71	2.298	5.283	−0.260	−0.795	0.64
Item 4	5.06	2.181	4.757	−0.065	−0.833	0.49
Item 5	5.58	2.142	4.588	−0.254	−0.803	0.60
Item 6	5.71	2.266	5.136	−0.179	−0.792	0.58
Item 8	5.66	2.354	5.539	−0.008	−0.826	0.50
*Compassion from others Action*						
Item 1	6.27	2.085	4.348	−0.340	−0.454	0.68
Item 2	5.92	2.119	4.492	−0.205	−0.520	0.65
Item 4	5.85	2.070	4.285	−0.221	−0.531	0.66
Item 5	6.86	2.197	4.828	−0.592	−0.398	0.65

*Note*. The values related to items 3 and 7 of the three Engagement subscales and the value related to item 3 of the three Action subscales were not calculated, as these were filler items.

### Confirmatory factor analysis of the factorial structure of the CEAS

CFA was performed in order to test the fit of a three-factor structure in line with the English and Portuguese original versions: Self-Compassion, Compassion from others, Compassion for others. Each of these factors was comprised of the Engagement and Actions dimensions. Therefore, the tested structure included six first-order factors and three second-order factors. The CFA produced unsatisfactory fit indices [χ2 (df) = 1358.396 (396), p ≤ 0.001; χ2/df = 3.43; CFI = 0.880; TLI = 0.868; RMSEA = 0.081 (0.0760.085); and SRMR = 0.068]. The analysis of the modification indices revealed the need to correlate certain residuals between items within each dimension. These links could be added because all the items shared similar wording [49].

**Fig 1:**
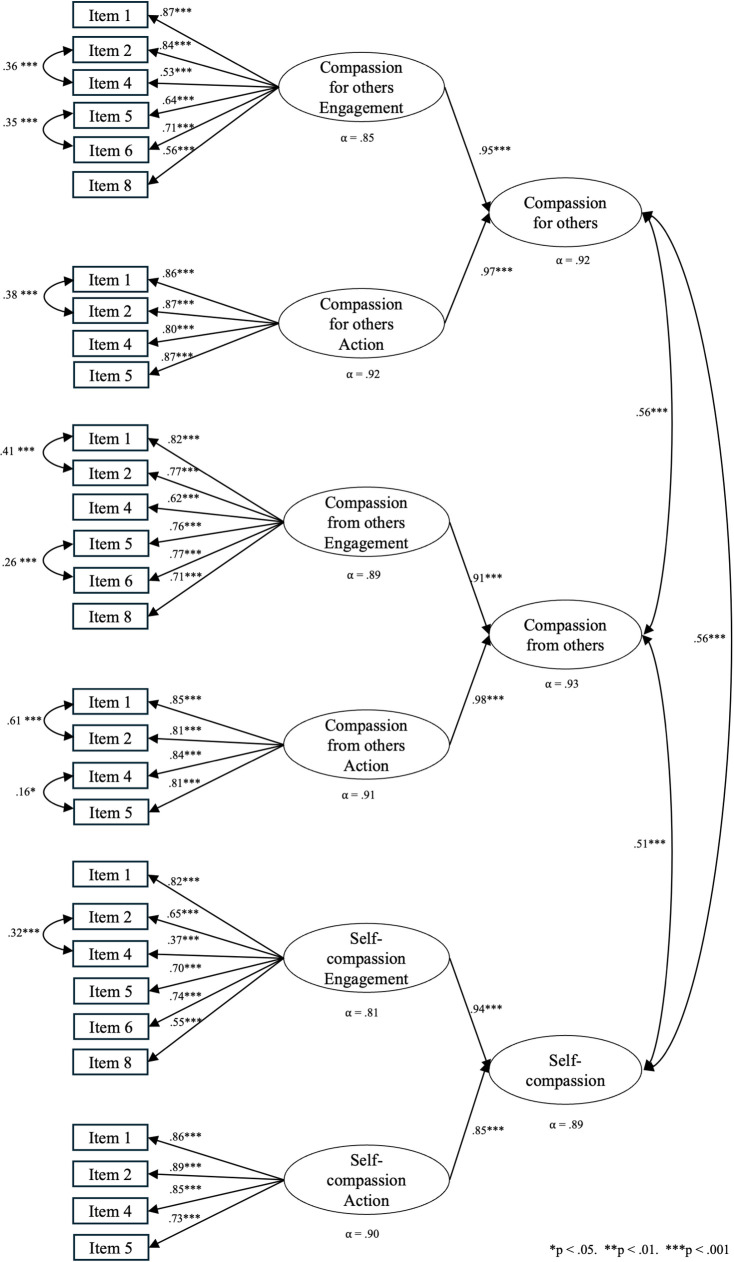
Path diagram from the CFA of the Italian version of the CEAS. Then, we checked if the CEAS scales were correlated with participants’ key sociodemographic variables, such as gender and age (see Table 4).

In particular, the errors of the following pairs of items were progressively correlated: for the Compassion from others scale, item 1 and 2 of both the engagement and action subscales, item 5 and 6 of the engagement scale, and item 4 and 5 of the action scale; for the Compassion for others scale, item 2 and 4, item 5 and 6 of the engagement subscale, item 1 and 2 of the action subscale. For the self-compassion scale, item 2 and 4 of the engagement subscale. After these modifications, the CFA model (Fig 1) was tested again in the same sample, yielding satisfactory fit indices [χ2 (df) = 1039.330 (388), p ≤ 0.001; χ2/df = 2.68; CFI = 0.919; TLI = 0.909; RMSEA = 0.067 (0.0620.072); and SRMR = 0.068]. The three compassion scales presented moderate inter-correlations. The three dimensions of the scale and their sub-dimensions had high reliability scores. As shown in Fig 1, Cronbach alpha was.89 for Self-compassion (Engagement alpha = .81; Action alpha = .90),.93 for Compassion from others (Engagement alpha = .89; Action alpha = .91) and.92 for Compassion for others (Engagement alpha = .85; Action alpha = .92).

**Table 4:** Correlations between CEAS and participants’ age and gender.

	CEAS Self-Compassion	CEAS Compassion for others	CEAS Compassion from others
Age	.109*	−.081	−.001
Gender	−.057	.058	−.046

Note. *p < .05.

As displayed in Table 4, all the correlations were weak. The highest was the one between CEAS self-compassion and participants’ age, which barely exceeded.10.

### Convergent and criterion-related validity

We went on examining the correlations between the CEAS and the other scales. As regards convergent validity (see Table 5), the CEAS Self-compassion scale had a medium positive correlation with self-compassion and self-reassuring measures, while it showed a moderate negative correlation with one of the two forms of self-criticism (Hated self). The correlation with the other form of self-criticism (Inadequate self) was negative and significant, but weaker. As expected, the Compassion for others scale showed a moderate positive correlation with empathy, while Compassion from others presented a strong correlation with the three forms of perceived social support. Taken together, these findings suggest that the three Compassion scales have good convergent validity.

**Table 5:** Convergent and criterion-related validity: correlations between variables.

	1	2	3	4	5	6	7	8	9	10	11	12
1.CEAS Self-compassion	1											
2.CEAS Compassion for others	.486^***^	1										
3.CEAS Compassion from others	.504^***^	.481^***^	1									
4.SCS Self-compassion scale	.451^***^	−0.050	.210^***^	1								
5.Empathy	.217^***^	.432^***^	.253^***^	.148^**^	1							
6.MSPSS Family	.148^**^	0.033	.321^***^	.206^***^	.172^***^	1						
7.MSPSS Friends	.249^***^	.246^***^	.516^***^	.121^*^	.279^***^	.318^***^	1					
8.MSPSS Significant others	.155^***^	.192^***^	.359^***^	0.090	.219^***^	.270^***^	.469^***^	1				
9.FSCRS Hated self	−.354^***^	0.027	−.240^***^	−.579^***^	−.165^***^	−.382^***^	−.215^***^	−.210^***^	1			
10.FSCRS Inadequate self	−.259^***^	.156^**^	−.142^**^	−.750^***^	−0.088	−.282^***^	−.117^*^	−0.087	.674^***^	1		
11.FSCRS_Self-reassuring	.502^***^	.116^*^	.295^***^	.712^***^	.219^***^	.230^***^	.185^***^	.179^***^	−.557^***^	−.527^***^	1	
12.PWB Pychological wellbeing	.423^***^	.174^***^	.281^***^	.517^***^	.303^***^	.241^***^	.350^***^	.269^***^	−.517^***^	−.468^***^	.575^***^	1
13.DASS	−.195^***^	.150^**^	−.137^**^	−.491^***^	−0.022	−.279^***^	−.149^**^	−0.031	.525^***^	.588^***^	−.395^***^	−.352^***^

Note. *p < .05, **p < .01, ***p < .001.

With respect to criterion-related validity (see Table 5), in line with our prediction, the Self-compassion and Compassion from others scales presented negative weak correlations with general distress, as measured by the DASS-21. Surprisingly, the Compassion for others scale was positively, although weakly, correlated with this variable. All the three scales were positively correlated with participants’ wellbeing, although the association presented by Self-compassion was moderate, while the others were weak.

## Discussion

The present study translated and adapted the CEAS into Italian. Findings offer support for the Italian version of the CEAS as a valid and reliable measure to assess the three flows of compassion: self-compassion, compassion for others and receptiveness to the compassion from others. Our findings are consistent with the structure found in the original study [2] in that each of the three scales can be used as single-factor scales or as separate engagement and action factors. Differently from previous studies, no item emerged as problematic. Item 4, which had to be removed in the Swedish and Portuguese versions for adolescents [12; 21], displayed both satisfactory correlation with the scale score and good factor loading on the corresponding factor.

The three scales had good internal consistency, indicating that they are reliable measures both when used as total scores and when used as subscales. In our study the three scales replicated the bifactorial structure for each compassion flow as found from the English- and Portuguese-speaking participants. Although the three flows were moderately correlated each flow also contains some specificity that is not shared with the others. This provides further confirmation that each flow represents a distinct process and focus for compassion. The CEAS convergent validity was good, as suggested by the correlations with other validated measures. The self-compassion scale of the CEAS was only moderately related to the Self-compassion scale by Neff at.451 [27]. This suggests that the two scales, which are built on different theoretical frameworks, assess different aspects of compassion for self. The CEAS self-compassion scale presented a positive and stronger association with self-reassurance, while it was negatively related to self-criticism, suggesting that it is linked with the ability to be supportive towards oneself.

In line with our prediction, compassion for others presented a moderate correlation with empathy, which confirms that this scale properly assesses the ability of being open and moved by the suffering of others. Finally, compassion from others was moderately correlated with perceived social support from family, friends, and significant others, which confirms that this scale assesses one’s ability to perceive other people as compassionate and receive support, care and kindness from them.

Regarding criterion-related validity, our findings partially confirmed our prediction. In line with literature [2,27], self-compassion is correlated with health-related outcomes, suggesting that being sensitive to one’s suffering and taking actions to prevent and alleviate it, positively contributes to individuals’ psychological functioning. Also the other two compassion flows played a role, but their contribution was minor. Notably, contrary to our prediction, we found that compassion for others was positively related to symptoms of depression, anxiety and stress. Such an unexpected result might suggest that being receptive and open to the suffering of others, combine with actions aimed at alleviating it, might be overwhelming, including a cost for oneself [2]. This focus on others could deprive individuals of relevant resources they could direct to themselves, thus limiting their levels of well-being. This explanation is in line with previous research findings concerning the link between empathy, depressive symptoms and anxiety, which indicated that emotions shared with other people may be overwhelming and stressful, thus increasing negative emotions, such as feelings of personal failure, helplessness, and guilt [50]. Empathic reactions to others might contribute to excessive interpersonal guilt in response to others’ distress, which in turn can facilitate the emergence of internalizing problems, such as depressive symptoms [51]. Anyway, the size of this correlation was small, so that its clinical relevance was limited. Such a relationship between compassion for others and mental health should be further investigated.

Some limitations of the present study should be acknowledged. First, our sample was a convenience one and was not representative of the general Italian population, although participants came from different Italian regions. Second, we did not examine the CEAS invariance across some relevant variables, such as age. Indeed, our sample consisted mainly of young adults, while older individuals were underrepresented. Given that older adults may have different compassion-related behaviors [e.g., 52], this age bias might limit the generalizability of our findings. Future studies could include a larger and more diverse sample, which would allow to gather insight into the equivalence of the scales in differently aged respondents. Third, the first model we tested produced unsatisfactory fit indices, so that we revised and tested it again among the same participants. We acknowledge that testing it on a separate validation sample would have given us greater assurance of its robustness. Finally, reliability was examined only in terms of internal consistency; further evidence of the CEAS reliability would be useful, especially in terms of the scales’ temporal stability. In this vein, future research could consider evaluating test-retest reliability of the scale or to perform a follow-up study.

Despite these limitations, the present study presents several strengths. Our findings show that the Italian version of the CEAS is a valid instrument that allows the detection of compassion by providing both an overall score and separate scores for each dimension. In this vein, our work extends the availability of the scale to a new linguistic group which may facilitate future cross-cultural comparisons. Moreover, this validation is particularly important for future research aimed at a better understanding of compassion in the Italian context, where there are no other specific scales for detecting compassion in its different flows.

In conclusion, we can state that the CEAS is a valid and reliable measure to assess different compassion orientations among Italian-speaking individuals. Given that compassion is the focus of research and intervention in the field of wellbeing and mental health also in Italy [53], it is important that researchers and health professionals can use instruments like the CEAS in order to detect compassion flows distinguishing between the engagement and action dimensions. Such an instrument can be used to both assess individuals’ needs and plan tailored programs, and to examine the efficacy of compassion based interventions.

## Supporting information

S1 Appendix The Italian version of the Compassionate Engagement and Action Scales. (DOCX)

## References

[1] Gilbert P. The origins and nature of compassion focused therapy. Br J Clin Psychol. 2014;53(1):6–41. doi: 10.1111/bjc.12043 24588760

[2] Gilbert P, Catarino F, Duarte C, Matos M, Kolts R, Stubbs J, et al. The development of compassionate engagement and action scales for self and others. J of Compassionate Health Care. 2017;4(1). doi: 10.1186/s40639-017-0033-3

[3] Gilbert P. Threat, safety, safeness and social safeness 30 years on: Fundamental dimensions and distinctions for mental health and well-being. Br J Clin Psychol. 2024;63(3):453–71. doi: 10.1111/bjc.12466 38698734

[4] Gilbert P, Simos G. Compassion Focused Therapy: Clinical practice and applications. London: Routledge. 2022.

[5] Gilbert P. Compassion focused therapy as an evolution informed, biopsychosocial science of the mind: History and challenge. In: Gilbert P, Simos G, editors. Compassion Focused Therapy: Clinical practice and applications. London: Routledge. 2022. p. 24–89.

[6] Gilbert P, Van Gordon W. Compassion as a Skill: A Comparison of Contemplative and Evolution-Based Approaches. Mindfulness. 2023;14(10):2395–416. doi: 10.1007/s12671-023-02173-w

[7] Sirois FM, Kitner R, Hirsch JK. Self-compassion, affect, and health-promoting behaviors. Health Psychol. 2015;34(6):661–9. doi: 10.1037/hea0000158 25243717

[8] Steindl SR, Kirby JN, Tellegan C. Motivational interviewing in compassion‐based interventions: Theory and practical applications. Clinical Psychol. 2018;22(3):265–79. doi: 10.1111/cp.12146

[9] Ramalho T, Pereira J, Ferreira C. How compassionate abilities influence the experience of loneliness and quality of life of people with and without chronic physical disease? J Psychol. 2021;155(8):679–94. doi: 10.1080/00223980.2021.1952922 34410887

[10] Saarinen AIL, Keltikangas-Järvinen L, Pulkki-Råback L, Cloninger CR, Elovainio M, Lehtimäki T, et al. The relationship of dispositional compassion with well-being: a study with a 15-year prospective follow-up. J Positive Psychol. 2019;15(6):806–20. doi: 10.1080/17439760.2019.1663251

[11] Ferreira C, Barreto M, Oliveira S. The Link Between Major Life Events and Quality of Life: The Role of Compassionate Abilities. Community Ment Health J. 2020;57(2):219–27. doi: 10.1007/s10597-020-00638-z 32440797

[12] Cunha M, Galhardo A, Gilbert P, Rodrigues C, Matos M. The flows of compassion in adolescents as measured by the compassionate engagement and action scales. Curr Psychol. 2021;42(9):7737–51. doi: 10.1007/s12144-021-02097-5

[13] Hermanto N, Zuroff DC. The social mentality theory of self-compassion and self-reassurance: The interactive effect of care-seeking and caregiving. J Soc Psychol. 2016;156(5):523–35. doi: 10.1080/00224545.2015.1135779 26736073

[14] Crocker J, Canevello A. Creating and undermining social support in communal relationships: the role of compassionate and self-image goals. J Pers Soc Psychol. 2008;95(3):555–75. doi: 10.1037/0022-3514.95.3.555 18729694

[15] Lee EE, Govind T, Ramsey M, Wu TC, Daly R, Liu J, et al. Compassion toward others and self-compassion predict mental and physical well-being: a 5-year longitudinal study of 1090 community-dwelling adults across the lifespan. Transl Psychiatry. 2021;11(1):397. doi: 10.1038/s41398-021-01491-8 34282145 PMC8287292

[16] Matos M, Palmeira L, Albuquerque I, Cunha M, Pedroso Lima M, Galhardo A, et al. Building Compassionate Schools: Pilot Study of a Compassionate Mind Training Intervention to Promote Teachers’ Well-being. Mindfulness. 2021;13(1):145–61. doi: 10.1007/s12671-021-01778-3

[17] Hermanto N, Zuroff DC, Kopala-Sibley DC, Kelly AC, Matos M, Gilbert P, et al. Ability to receive compassion from others buffers the depressogenic effect of self-criticism: A cross-cultural multi-study analysis. Personality Individual Differences. 2016;98:324–32. doi: 10.1016/j.paid.2016.04.055

[18] Gilbert P. Compassion: From Its Evolution to a Psychotherapy. Front Psychol. 2020;11:586161. doi: 10.3389/fpsyg.2020.586161 33362650 PMC7762265

[19] Asano K, Kotera Y, Tsuchiya M, Ishimura I, Lin S, Matsumoto Y, et al. The development of the Japanese version of the compassionate engagement and action scales. PLoS One. 2020;15(4):e0230875. doi: 10.1371/journal.pone.0230875 32236112 PMC7112184

[20] Leboeuf I, Andreotti E, Irons C, Beaumont E, Antoine P. A Randomized Controlled Study of a French Compassionate Mind Training. Mindfulness. 2022;13(11):2891–903. doi: 10.1007/s12671-022-01987-4

[21] Henje E, Rindestig FC, Gilbert P, Dennhag I. Psychometric validity of the Compassionate Engagement and Action Scale for Adolescents: a Swedish version. Scand J Child Adolesc Psychiatr Psychol. 2020;8:70–80. doi: 10.21307/sjcapp-2020-007 33520779 PMC7685496

[22] Halamová J, Kanovský M, Pacúchová M. Psychometric analysis of the slovak version of the compassionate engagement and action scales. J Psychological Educational Res. 2020;28(1):64–80.

[23] Davalos-Batallas V, Vargas-Martínez A-M, Bonilla-Sierra P, Leon-Larios F, Lomas-Campos M-L-M, Vaca-Gallegos S-L, et al. Compassionate Engagement and Action in the Education for Health Care Professions: A Cross-Sectional Study at an Ecuadorian University. Int J Environ Res Public Health. 2020;17(15):5425. doi: 10.3390/ijerph17155425 32731430 PMC7432900

[24] Ari E, Cesur-Soysal G, Basran J, Gilbert P. The Compassionate Engagement and Action Scales for Self and Others: Turkish Adaptation, Validity, and Reliability Study. Front Psychol. 2022;13:780077. doi: 10.3389/fpsyg.2022.780077 35496178 PMC9046699

[25] Brophy K, Emery M, MacDonald C, Côté CI, Körner A. Validation of the compassionate engagement and action scales, compassion scale, and Sussex-Oxford compassion scales in a French-Canadian sample. PLoS One. 2024;19(6):e0305776. doi: 10.1371/journal.pone.0305776 38913657 PMC11195958

[26] Petrocchi N, Ottaviani C, Couyoumdjian A. Dimensionality of self-compassion: translation and construct validation of the self-compassion scale in an Italian sample. J Ment Health. 2014;23(2):72–7. doi: 10.3109/09638237.2013.841869 24328923

[27] Neff KD. The Development and Validation of a Scale to Measure Self-Compassion. Self and Identity. 2003;2(3):223–50. doi: 10.1080/15298860309027

[28] Neff KD. Erratum to: The Self-Compassion Scale is a Valid and Theoretically Coherent Measure of Self-Compassion. Mindfulness. 2016;7(4):1009. doi: 10.1007/s12671-016-0560-6

[29] International Test Commission. The ITC Guidelines for Translating and Adapting Tests. 2017. https://www.intestcom.org/files/guideline_test_adaptation_2ed.pdf

[30] Petrocchi N, Couyoumdjian A. The impact of gratitude on depression and anxiety: the mediating role of criticizing, attacking, and reassuring the self. Self and Identity. 2015;15(2):191–205. doi: 10.1080/15298868.2015.1095794

[31] Gilbert P, Clarke M, Hempel S, Miles JNV, Irons C. Criticizing and reassuring oneself: An exploration of forms, styles and reasons in female students. Br J Clin Psychol. 2004;43(Pt 1):31–50. doi: 10.1348/014466504772812959 15005905

[32] Capozza D, Trifiletti E, Vezzali L, Favara I. Can intergroup contact improve humanity attributions?. Int J Psychol. 2013;48(4):527–41. doi: 10.1080/00207594.2012.688132 22721357

[33] Prezza M, Principato MC. La rete sociale e il sostegno sociale. In: Prezza M, Santinello M, editors. Conoscere la comunità. Bologna: Il Mulino. 2002. p. 193–233.

[34] Zimet GD, Dahlem NW, Zimet SG, Farley GK. The Multidimensional Scale of Perceived Social Support. Journal of Personality Assessment. 1988;52(1):30–41. doi: 10.1207/s15327752jpa5201_2 2280326

[35] Sirigatti S, Stefanile C, Giannetti E, Iani L, Penzo I, Mazzeschi A. Assessment of factor structure of Ryff’s Psychological Well-Being Scales in Italian adolescents. Bollettino di Psicologia Applicata. 2009;259:30–50.

[36] Ryff CD, Keyes CL. The structure of psychological well-being revisited. J Pers Soc Psychol. 1995;69(4):719–27. doi: 10.1037//0022-3514.69.4.719 7473027

[37] Bottesi G, Ghisi M, Altoè G, Conforti E, Melli G, Sica C. The Italian version of the Depression Anxiety Stress Scales-21: Factor structure and psychometric properties on community and clinical samples. Compr Psychiatry. 2015;60:170–81. doi: 10.1016/j.comppsych.2015.04.005 25933937

[38] Henry JD, Crawford JR. The short-form version of the Depression Anxiety Stress Scales (DASS-21): construct validity and normative data in a large non-clinical sample. Br J Clin Psychol. 2005;44(Pt 2):227–39. doi: 10.1348/014466505X29657 16004657

[39] West SG, Finch JF, Curran PJ. Structural equation models with nonnormal variables: Problems and remedies. In: Hoyle FH, editor. Structural equation modeling: Concepts, issues, and applications. Sage Publications, Inc. 1995. p. 56–75.

[40] Nunnally JC, Bernstein IH. Psychometric theory. New York: McGraw-Hill. 1994.

[41] Muthén LK, Muthén BO. Mplus: Statistical Analysis with Latent Variables: User’s Guide. 8 ed. Los Angeles, CA: Authors. 2017.

[42] Hu L, Bentler PM. Cutoff criteria for fit indexes in covariance structure analysis: Conventional criteria versus new alternatives. Structural Equation Modeling: A Multidisciplinary Journal. 1999;6(1):1–55. doi: 10.1080/10705519909540118

[43] Hayes AF. Introduction to Mediation, Moderation, and Conditional Process Analysis: A Regression-Based Perspective. New York, USA: The Guilford Press. 2018.

[44] McNeish D, An J, Hancock GR. The thorny relation between measurement quality and fit index cutoffs in latent variable models. J Pers Assess. 2018;100(1):43–52. doi: 10.1080/00223891.2017.1281286 28631976

[45] Ullman JB. Structural equation modeling. In: Tabachnick BG, Fidell LS, editors. Using multivariate statistics. Boston, MA: Pearson Education. 2001. p. 653–771.

[46] Pan J, Ip EH, Dubé L. An alternative to post hoc model modification in confirmatory factor analysis: The Bayesian lasso. Psychol Methods. 2017;22(4):687–704. doi: 10.1037/met0000112 29265848 PMC5745070

[47] Brown TA. Confirmatory factor analysis for applied research. Guilford Press. 2006.

[48] Cohen J. Statistical power analysis for the behavioral sciences. 2nd ed. Hillsdale, NJ: Lawrence Erlbaum Associates, Publishers. 1988.

[49] Green SB, Hershberger SL. Correlated Errors in True Score Models and Their Effect on Coefficient Alpha. Structural Equation Modeling: A Multidisciplinary J. 2000;7(2):251–70. doi: 10.1207/s15328007sem0702_6

[50] Gambin M, Sharp C. Relations between empathy and anxiety dimensions in inpatient adolescents. Anxiety Stress Coping. 2018;31(4):447–58. doi: 10.1080/10615806.2018.1475868 29772912

[51] Tone EB, Tully EC. Empathy as a “risky strength”: a multilevel examination of empathy and risk for internalizing disorders. Dev Psychopathol. 2014;26(4 Pt 2):1547–65. doi: 10.1017/S0954579414001199 25422978 PMC4340688

[52] Tavares L, Xavier A, Vagos P, Castilho P, Cunha M, Pinto-Gouveia J. Lifespan perspective on self-compassion: Insights from age-groups and gender comparisons. Appl Devel Sci. 2024;1–17. doi: 10.1080/10888691.2024.2432864

[53] Petrocchi N, Cosentino T, Pellegrini V, Femia G, D’Innocenzo A, Mancini F. Compassion-focused group therapy for treatment-resistant ocd: initial evaluation using a multiple baseline design. Front Psychol. 2021;11:594277. doi: 10.3389/fpsyg.2020.594277 33510677 PMC7835278

