# Supplementary material for: Assessing compassionate abilities: Translation and psychometric properties of the Italian version of the compassionate engagement and action scales (CEAS)
Source: PLoS One. 2025 Jul 15;20(7):e0326922. doi: 10.1371/journal.pone.0326922 (PMC12262902; doi:10.1371/journal.pone.0326922)
Supplement: S1 Appendix — (DOCX) [file pone.0326922.s001.docx]

**Appendix**

*The Italian version of the Compassionate Engagement and Action Scales*

Definiamo la compassione come "la sensibilità alla nostra e all’altrui sofferenza con l'impegno di cercare di alleviarla e prevenirla". Questo significa che ci sono due aspetti della compassione. Il *primo* è la capacità di essere motivati a entrare in contatto con sentimenti difficili, invece di cercare di evitarli o sopprimerli. Il *secondo* aspetto della compassione è la capacità di concentrarsi su ciò che è d’aiuto per noi. Proprio come un medico con il suo paziente. Il primo aspetto è essere motivati e capaci di prestare attenzione al dolore e (imparare come) dargli un senso. Il secondo è essere in grado di intraprendere l’azione che potrà essere d’aiuto. Di seguito troverà una serie di domande che riguardano questi due aspetti della compassione.

Quando le cose ci vanno male e ci sentiamo sofferenti a causa di battute d'arresto, fallimenti, delusioni o perdite, possiamo affrontarle in modi diversi. Siamo interessati al grado in cui le persone possono essere **compassionevoli con se stesse**. Perciò legga attentamente ogni affermazione e pensi a come si applica a Lei quando soffre. Risponda ad ogni affermazione utilizzando una scala che va da 1 a 10, considerando che 1 corrisponde a mai e 10 corrisponde a sempre.

Le seguenti domande riguardano quanto Lei è motivato e capace di stare a contatto con la Sua sofferenza quando la sperimenta. Quindi:

quando sono sofferente o turbato da qualcosa…

|  |  | **Mai** |  |  |  |  |  |  |  |  | **Sempre** |
| --- | --- | --- | --- | --- | --- | --- | --- | --- | --- | --- | --- |
| 01. | Sono motivato ad occuparmi e affrontare la mia sofferenza quando si presenta. | 🞏_1_ | 🞏_2_ | 🞏_3_ | 🞏_4_ | 🞏_5_ | 🞏_6_ | 🞏_7_ | 🞏_8_ | 🞏_9_ | 🞏_10_ |
| 02. | Noto e sono sensibile alla mia sofferenza quando si presenta. | 🞏_1_ | 🞏_2_ | 🞏_3_ | 🞏_4_ | 🞏_5_ | 🞏_6_ | 🞏_7_ | 🞏_8_ | 🞏_9_ | 🞏_10_ |
| 03. | Evito di pensare alla mia sofferenza, provo a distrarmi e a scacciarla dalla mia testa. | 🞏_1_ | 🞏_2_ | 🞏_3_ | 🞏_4_ | 🞏_5_ | 🞏_6_ | 🞏_7_ | 🞏_8_ | 🞏_9_ | 🞏_10_ |
| 04. | Sono emotivamente commosso dai sentimenti e dalle situazioni di sofferenza. | 🞏_1_ | 🞏_2_ | 🞏_3_ | 🞏_4_ | 🞏_5_ | 🞏_6_ | 🞏_7_ | 🞏_8_ | 🞏_9_ | 🞏_10_ |
| 05. | Tollero i diversi sentimenti che fanno parte della mia sofferenza. | 🞏_1_ | 🞏_2_ | 🞏_3_ | 🞏_4_ | 🞏_5_ | 🞏_6_ | 🞏_7_ | 🞏_8_ | 🞏_9_ | 🞏_10_ |
| 06. | Rifletto e attribuisco un senso alla mia sofferenza. | 🞏_1_ | 🞏_2_ | 🞏_3_ | 🞏_4_ | 🞏_5_ | 🞏_6_ | 🞏_7_ | 🞏_8_ | 🞏_9_ | 🞏_10_ |
| 07. | Non sopporto di soffrire. | 🞏_1_ | 🞏_2_ | 🞏_3_ | 🞏_4_ | 🞏_5_ | 🞏_6_ | 🞏_7_ | 🞏_8_ | 🞏_9_ | 🞏_10_ |
| 08. | Accetto, non critico e non giudico la mia sofferenza. | 🞏_1_ | 🞏_2_ | 🞏_3_ | 🞏_4_ | 🞏_5_ | 🞏_6_ | 🞏_7_ | 🞏_8_ | 🞏_9_ | 🞏_10_ |

Le seguenti domande riguardano come Lei risponde attivamente in modo compassionevole alle emozioni, ai pensieri e alle situazioni che Le causano sofferenza. Quindi:

quando sono sofferente o turbato da qualcosa…

|  |  | **Mai** |  |  |  |  |  |  |  |  | **Sempre** |
| --- | --- | --- | --- | --- | --- | --- | --- | --- | --- | --- | --- |
| 01. | Dirigo la mia attenzione su ciò che probabilmente può essermi d'aiuto. | 🞏_1_ | 🞏_2_ | 🞏_3_ | 🞏_4_ | 🞏_5_ | 🞏_6_ | 🞏_7_ | 🞏_8_ | 🞏_9_ | 🞏_10_ |
| 02. | Penso e trovo soluzioni utili per affrontare la mia sofferenza. | 🞏_1_ | 🞏_2_ | 🞏_3_ | 🞏_4_ | 🞏_5_ | 🞏_6_ | 🞏_7_ | 🞏_8_ | 🞏_9_ | 🞏_10_ |
| 03. | Non so come aiutare me stesso. | 🞏_1_ | 🞏_2_ | 🞏_3_ | 🞏_4_ | 🞏_5_ | 🞏_6_ | 🞏_7_ | 🞏_8_ | 🞏_9_ | 🞏_10_ |
| 04. | Intraprendo azioni e faccio ciò che potrà essermi utile. | 🞏_1_ | 🞏_2_ | 🞏_3_ | 🞏_4_ | 🞏_5_ | 🞏_6_ | 🞏_7_ | 🞏_8_ | 🞏_9_ | 🞏_10_ |
| 05. | Assumo un atteggiamento di supporto, disponibilità e incoraggiamento verso me stesso. | 🞏_1_ | 🞏_2_ | 🞏_3_ | 🞏_4_ | 🞏_5_ | 🞏_6_ | 🞏_7_ | 🞏_8_ | 🞏_9_ | 🞏_10_ |

Quando le cose vanno male alle altre persone e queste diventano sofferenti a causa di battute d'arresto, fallimenti, delusioni o perdite, possiamo affrontare la loro sofferenza in modi diversi. Siamo interessati al grado in cui le persone possono essere **compassionevoli con gli altri**. Perciò legga attentamente ogni affermazione e pensi a come ciascuna si applica a Lei quando le **persone che fanno parte della sua vita** soffrono. Risponda ad ogni affermazione utilizzando una scala che va da 1 a 10, considerando che 1 corrisponde a mai e 10 corrisponde a sempre.

Le seguenti domande riguardano quanto Lei è motivato e capace di stare a contatto con la sofferenza di altre persone quando questi la sperimentano. Quindi:

quando gli altri sono sofferenti o turbati da qualcosa…

|  |  | **Mai** |  |  |  |  |  |  |  |  | **Sempre** |
| --- | --- | --- | --- | --- | --- | --- | --- | --- | --- | --- | --- |
| 01. | Sono motivato ad occuparmi e affrontare la sofferenza degli altri quando si presenta. | 🞏_1_ | 🞏_2_ | 🞏_3_ | 🞏_4_ | 🞏_5_ | 🞏_6_ | 🞏_7_ | 🞏_8_ | 🞏_9_ | 🞏_10_ |
| 02. | Noto e sono sensibile alla sofferenza degli altri quando si presenta. | 🞏_1_ | 🞏_2_ | 🞏_3_ | 🞏_4_ | 🞏_5_ | 🞏_6_ | 🞏_7_ | 🞏_8_ | 🞏_9_ | 🞏_10_ |
| 03. | Evito di pensare alla sofferenza degli altri, provo a distrarmi e a scacciarla dalla mia testa. | 🞏_1_ | 🞏_2_ | 🞏_3_ | 🞏_4_ | 🞏_5_ | 🞏_6_ | 🞏_7_ | 🞏_8_ | 🞏_9_ | 🞏_10_ |
| 04. | Sono emotivamente commosso dalle espressioni di sofferenza degli altri. | 🞏_1_ | 🞏_2_ | 🞏_3_ | 🞏_4_ | 🞏_5_ | 🞏_6_ | 🞏_7_ | 🞏_8_ | 🞏_9_ | 🞏_10_ |
| 05. | Tollero i diversi sentimenti che fanno parte della sofferenza degli altri. | 🞏_1_ | 🞏_2_ | 🞏_3_ | 🞏_4_ | 🞏_5_ | 🞏_6_ | 🞏_7_ | 🞏_8_ | 🞏_9_ | 🞏_10_ |
| 06. | Rifletto e attribuisco un senso alla sofferenza degli altri. | 🞏_1_ | 🞏_2_ | 🞏_3_ | 🞏_4_ | 🞏_5_ | 🞏_6_ | 🞏_7_ | 🞏_8_ | 🞏_9_ | 🞏_10_ |
| 07. | Non sopporto la sofferenza degli altri. | 🞏_1_ | 🞏_2_ | 🞏_3_ | 🞏_4_ | 🞏_5_ | 🞏_6_ | 🞏_7_ | 🞏_8_ | 🞏_9_ | 🞏_10_ |
| 08. | Accetto, non critico e non giudico la sofferenza degli altri. | 🞏_1_ | 🞏_2_ | 🞏_3_ | 🞏_4_ | 🞏_5_ | 🞏_6_ | 🞏_7_ | 🞏_8_ | 🞏_9_ | 🞏_10_ |

Le seguenti domande riguardano come Lei risponde attivamente in modo compassionevole alla sofferenza delle altre persone. Quindi:

quando gli altri sono sofferenti o turbati da qualcosa…

|  |  | **Mai** |  |  |  |  |  |  |  |  | **Sempre** |
| --- | --- | --- | --- | --- | --- | --- | --- | --- | --- | --- | --- |
| 01. | Dirigo la mia attenzione su ciò che probabilmente può essere d'aiuto agli altri. | 🞏_1_ | 🞏_2_ | 🞏_3_ | 🞏_4_ | 🞏_5_ | 🞏_6_ | 🞏_7_ | 🞏_8_ | 🞏_9_ | 🞏_10_ |
| 02. | Penso e trovo soluzioni utili per le altre persone per affrontare la loro sofferenza. | 🞏_1_ | 🞏_2_ | 🞏_3_ | 🞏_4_ | 🞏_5_ | 🞏_6_ | 🞏_7_ | 🞏_8_ | 🞏_9_ | 🞏_10_ |
| 03. | Non so come aiutare le altre persone quando soffrono. | 🞏_1_ | 🞏_2_ | 🞏_3_ | 🞏_4_ | 🞏_5_ | 🞏_6_ | 🞏_7_ | 🞏_8_ | 🞏_9_ | 🞏_10_ |
| 04. | Intraprendo le azioni e faccio ciò che potrà essere utile per gli altri. | 🞏_1_ | 🞏_2_ | 🞏_3_ | 🞏_4_ | 🞏_5_ | 🞏_6_ | 🞏_7_ | 🞏_8_ | 🞏_9_ | 🞏_10_ |
| 05. | Esprimo sentimenti di supporto, disponibilità e incoraggiamento verso gli altri. | 🞏_1_ | 🞏_2_ | 🞏_3_ | 🞏_4_ | 🞏_5_ | 🞏_6_ | 🞏_7_ | 🞏_8_ | 🞏_9_ | 🞏_10_ |

Quando le cose ci vanno male e ci sentiamo sofferenti a causa di battute d'arresto, fallimenti, delusioni o perdite, gli altri possono affrontare la nostra sofferenza in modi diversi. Siamo interessati al grado in cui ritiene che le **persone importanti nella Sua vita** possano essere compassionevoli nei confronti della Sua sofferenza. Perciò legga attentamente ogni affermazione e pensi a come si applica alle **persone importanti della Sua vita** quando Lei soffre. Risponda ad ogni affermazione utilizzando una scala che va da 1 a 10, considerando che 1 corrisponde a mai e 10 corrisponde a sempre.

Le seguenti domande riguardano quanto Lei pensa che gli altri siano motivati a stare in contatto e quanto stiano a contatto con la Sua sofferenza quando Lei la sperimenta. Quindi:

quando sono sofferente o turbato da qualcosa…

|  |  | **Mai** |  |  |  |  |  |  |  |  | **Sempre** |
| --- | --- | --- | --- | --- | --- | --- | --- | --- | --- | --- | --- |
| 01. | Gli altri sono motivati ad occuparsi e affrontare la mia sofferenza quando si presenta. | 🞏_1_ | 🞏_2_ | 🞏_3_ | 🞏_4_ | 🞏_5_ | 🞏_6_ | 🞏_7_ | 🞏_8_ | 🞏_9_ | 🞏_10_ |
| 02. | Gli altri notano e sono sensibili alla mia sofferenza quando si presenta. | 🞏_1_ | 🞏_2_ | 🞏_3_ | 🞏_4_ | 🞏_5_ | 🞏_6_ | 🞏_7_ | 🞏_8_ | 🞏_9_ | 🞏_10_ |
| 03. | Gli altri evitano di pensare alla mia sofferenza, provano a distrarsi e a scacciarla dalla loro testa. | 🞏_1_ | 🞏_2_ | 🞏_3_ | 🞏_4_ | 🞏_5_ | 🞏_6_ | 🞏_7_ | 🞏_8_ | 🞏_9_ | 🞏_10_ |
| 04. | Gli altri sono emotivamente commossi dalla mia sofferenza. | 🞏_1_ | 🞏_2_ | 🞏_3_ | 🞏_4_ | 🞏_5_ | 🞏_6_ | 🞏_7_ | 🞏_8_ | 🞏_9_ | 🞏_10_ |
| 05. | Gli altri tollerano i diversi sentimenti che fanno parte della mia sofferenza. | 🞏_1_ | 🞏_2_ | 🞏_3_ | 🞏_4_ | 🞏_5_ | 🞏_6_ | 🞏_7_ | 🞏_8_ | 🞏_9_ | 🞏_10_ |
| 06. | Gli altri riflettono e attribuiscono un senso alla mia sofferenza. | 🞏_1_ | 🞏_2_ | 🞏_3_ | 🞏_4_ | 🞏_5_ | 🞏_6_ | 🞏_7_ | 🞏_8_ | 🞏_9_ | 🞏_10_ |
| 07. | Gli altri non sopportano la mia sofferenza. | 🞏_1_ | 🞏_2_ | 🞏_3_ | 🞏_4_ | 🞏_5_ | 🞏_6_ | 🞏_7_ | 🞏_8_ | 🞏_9_ | 🞏_10_ |
| 08. | Gli altri accettano, non criticano e non giudicano la mia sofferenza. | 🞏_1_ | 🞏_2_ | 🞏_3_ | 🞏_4_ | 🞏_5_ | 🞏_6_ | 🞏_7_ | 🞏_8_ | 🞏_9_ | 🞏_10_ |

Le seguenti domande riguardano come gli altri rispondono attivamente in modo compassionevole alle emozioni e alle situazioni che Le causano sofferenza. Quindi:

quando sono sofferente o turbato da qualcosa…

|  |  | **Mai** |  |  |  |  |  |  |  |  | **Sempre** |
| --- | --- | --- | --- | --- | --- | --- | --- | --- | --- | --- | --- |
| 01. | Gli altri dirigono la loro attenzione su ciò che probabilmente può essermi d'aiuto. | 🞏_1_ | 🞏_2_ | 🞏_3_ | 🞏_4_ | 🞏_5_ | 🞏_6_ | 🞏_7_ | 🞏_8_ | 🞏_9_ | 🞏_10_ |
| 02. | Gli altri pensano e trovano soluzioni utili per me per affrontare la mia sofferenza. | 🞏_1_ | 🞏_2_ | 🞏_3_ | 🞏_4_ | 🞏_5_ | 🞏_6_ | 🞏_7_ | 🞏_8_ | 🞏_9_ | 🞏_10_ |
| 03. | Gli altri non sanno come aiutarmi quando soffro. | 🞏_1_ | 🞏_2_ | 🞏_3_ | 🞏_4_ | 🞏_5_ | 🞏_6_ | 🞏_7_ | 🞏_8_ | 🞏_9_ | 🞏_10_ |
| 04. | Gli altri intraprendono le azioni e fanno ciò che potrà essermi utile. | 🞏_1_ | 🞏_2_ | 🞏_3_ | 🞏_4_ | 🞏_5_ | 🞏_6_ | 🞏_7_ | 🞏_8_ | 🞏_9_ | 🞏_10_ |
| 05. | Gli altri mi rivolgono sentimenti di supporto, disponibilità e incoraggiamento. | 🞏_1_ | 🞏_2_ | 🞏_3_ | 🞏_4_ | 🞏_5_ | 🞏_6_ | 🞏_7_ | 🞏_8_ | 🞏_9_ | 🞏_10_ |
